# Design of high bulk moduli high entropy alloys using machine learning

**DOI:** 10.1038/s41598-023-47181-x

**Published:** 2023-11-22

**Authors:** Manjunadh Kandavalli, Abhishek Agarwal, Ansh Poonia, Modalavalasa Kishor, Kameswari Prasada Rao Ayyagari

**Affiliations:** 1 Nanyang Technological University, Singapore, 639798 Singapore; 2 BML Munjal University, Gurgaon, 122413 India

**Keywords:** Structural materials, Materials science, Computational methods

## Abstract

In this work, the authors have demonstrated the use of machine learning (ML) models in the prediction of bulk modulus for High Entropy Alloys (HEA). For the first time, ML has been used for optimizing the composition of HEA to achieve enhanced bulk modulus values. A total of 12 ML algorithms were trained to classify the elemental composition as HEA or non-HEA. Among these models, Gradient Boosting Classifier (GBC) was found to be the most accurate, with a test accuracy of 78%. Further, six regression models were trained to predict the bulk modulus of HEAs, and the best results were obtained by LASSO Regression model with an R-square value of 0.98 and an adjusted R-Square value of 0.97 for the test data set. This work effectively bridges the gap in the discovery and property analysis of HEAs. By accelerating material discovery via providing alternate means for designing virtual alloy compositions having favourable bulk modulus for respective applications, this work opens new avenues of applications of HEAs.

## Introduction

Conventional alloy systems usually have one principal element, with other elements in minimal concentrations. The alloy systems of brass and steel, which are copper, and iron-based respectively are two examples of conventional alloys. Researchers have developed a new set of alloys consisting of single-phase random solid solutions. These multi-component alloys, known as high entropy alloys (HEAs)^1^, are equiatomic and possess high configurational entropy. In the initial studies, multicomponent alloys containing 5–9 elements were investigated. Subsequently, later studies have considered HEAs with four elements, either equiatomic or non-equiatomic in nature^2,3^. Contrary to the conventional metallurgy knowledge and binary, tertiary, and ternary phase diagrams suggesting several kinds of phases and intermetallic compounds for multi-element alloys, experimental studies revealed a rather peculiar effect of reduction in the number of phases for alloys with higher mixing entropy values^4^. This kind of unique phase formation of HEAs are indicative of their exceptional properties and in turn, makes them useful in many applications. Also, due to multi-principal element composition, HEAs tend to display unique properties of their constituent elements like exceptional high-temperature strength, high hardness, better structural stability, great resistance to corrosion, oxidation, and weariness^5^. This kind of combination of properties in a particular alloy system is unusual in conventional alloys and hence designing and development of HEAs have been the centre of many research topics in the past few decades. This combination of properties has enabled discoveries of many potential applications of HEAs, such as gas turbines, nozzles, blades, compressors, automobile components, space crafts, missiles, submarines, and so on^6^.

Among all the properties of HEAs, one of the most notable properties of the HEA is its bulk modulus (k). The bulk modulus defines the ratio of the applied pressure (P) to the relative change in volume ($v$), which has been represented in Eq. (1). The applied pressure or hydrostatic force directly results in a change in volume without changing shape.

1 $$k=-v\frac{dP}{dv}$$

Materials having high bulk modulus make them suitable for applications that need to operate under a high magnitude of pressure from all sides, for instance, machines operating in deep ocean territories, e.g., submarines, pipelines, torpedoes, etc.^7^. Designing HEAs that have a high bulk modulus opens up an avenue in the field of HEAs. Bulk Modulus values give us a quantitative estimate of the compressibility of the alloy. It is well known that high bulk modulus corresponds to high strength^8,9^. In this study, we trained a regressor model that uses all the possible elemental compositions for a given set of elements as inputs to predict the respective bulk moduli values. The elemental composition corresponding to the maximum bulk modulus value (output of the model), will be a desired virtual HEA. Present work would further assist the scientific community in the accelerated discovery of HEAs based on their high bulk modulus applications and provide a sense of direction to the experimentalists, with an increased success rate.

With the increase in the efficiency of computational methodologies, many ML approaches have found their applications in HEA phase prediction and designing. Huang et al. developed a model which predicts the phase of HEA by elemental inputs using ML for around 400 HEA systems, with an accuracy of about 70%^10^. Zhang et al. focussed on optimising classification models to identify solid and non-solid solution HEAs and further using ML models to segregate them into face-centred-cubic (FCC), body-centred-cubic (BCC), and dual-phase HEAs^11^. Agarwal et al. predicted FCC and BCC phases in HEAs based on both their elemental composition and thermodynamic parameters through artificial intelligence^12^. Further, research work by Pyzer et. al. suggested that artificial intelligence can accelerate and enhance material discovery^13^. Kim et al.^14^ conducted a study to predict the elastic properties of Al0.3CoCrFeNi High Entropy Alloy (HEA) using first principles and ML models. For the prediction of bulk and shear moduli, they employed the gradient boosting trees algorithm. Concurrently, Density Functional Theory (DFT) calculations were performed using VASP. Additionally, Vickers hardness was calculated based on the empirical relation between bulk and shear moduli.

Roy et al.^15^ conducted a study in which they utilized gradient boost regression to predict Young's modulus of high entropy alloys (HEAs). The dataset used in the analysis consisted of 87 entries, and the model they developed achieved predictive accuracy with a Root Mean Square Error (RMSE) of 87.76%. To enhance the model's performance, the researchers also performed hyperparameter optimization. These findings provide valuable insights into the factors influencing Young's modulus of high entropy alloys and contribute to a better understanding of their mechanical behaviour. Variations in composition significantly affect the mechanical properties of materials and this knowledge space aids in developing alloys with the required properties. Zhang et al.^16^ conducted a study to predict the mechanical properties, such as yield strength and Young's modulus, for a non-equiatomic CuFeNiCrCo alloy. The study utilized molecular dynamics simulations (MD) in combination with ML models for property prediction. Among all the algorithms used, the Kernel-based extreme learning machine model outperformed all others, demonstrating superior accuracy in predicting the mechanical properties of the alloy. Mei et al.^17^ designed a method for predicting the elastic properties of refractory HEAs using the EMTO-CPA (Exact Muffin-Tin Orbital Method combined with Coherent Potential Approximation) and Random Forest model. By employing DFT approaches and the EMTO method, they developed 2467 elastic constants and utilized this dataset to predict the elastic properties using the Random Forest model.

Many other researchers have deployed ML techniques to predict the phases of HEAs, but the research has been limited to phase prediction and they fall short of demonstrating an exhaustive set of modeling techniques^18–22^. Research by Bhandari et al. focussed on yield strength prediction of high entropy alloys^23^, but to the author’s knowledge, there’s no HEA composition-based prediction on elements favouring certain properties, for example, bulk modulus in this work^24–28^. For the first time, ML has been used to optimize the composition for achieving higher values of Bulk modulus for the HEA systems. This work presents the scope of designing HEAs having a wide range of bulk moduli values, for a given set of elements by predicting the optimum proportions of each element in a HEA. From this set, the HEA corresponding to the highest bulk modulus value can be selected for high-strength applications by the user.

## Methodology

### Pre-processing

In the model, the elements from a given set and final required phases of HEA are taken as the input parameters and then, the algorithm, through a combination of ML models (HEA classifier and BM regressor), predicts the most favourable elemental composition to optimize the bulk-modulus for the HEA. The HEA classifier was trained by using data from the research work of Dam et al.^29^ and Reddy et al.^30^. The data used for the prediction of bulk modulus values for the HEA cluster was taken from Precker et al.^31^ and Mishra et al.^32^.

The raw data was pre-processed before using in the work. The first step was to extract the constituent elements of the alloys from raw data and their number of moles were co-related with HEA forming ability of the alloy. HEAs were labelled as 1 and non-HEAs as 0. In the next step, phase data is converted into a binary representation vector to be used as input. After pre-processing it was found that a sub-group of 37 elements (from 79 elements present in the dataset) prominently form HEAs in various combinations. The authors have tried to cover the maximum number of elements that are commonly used to predict HEAs, but also acknowledge the fact that the model can be extended to include new elements which are found to form HEAs. Constituent elements of HEAs are a good indicator to establish a correlation between composition and their phases, as demonstrated by Agarwal et al.^12^. Therefore, in this work, the elemental composition based on the number of moles was used for HEA classification.

**Figure 1:**
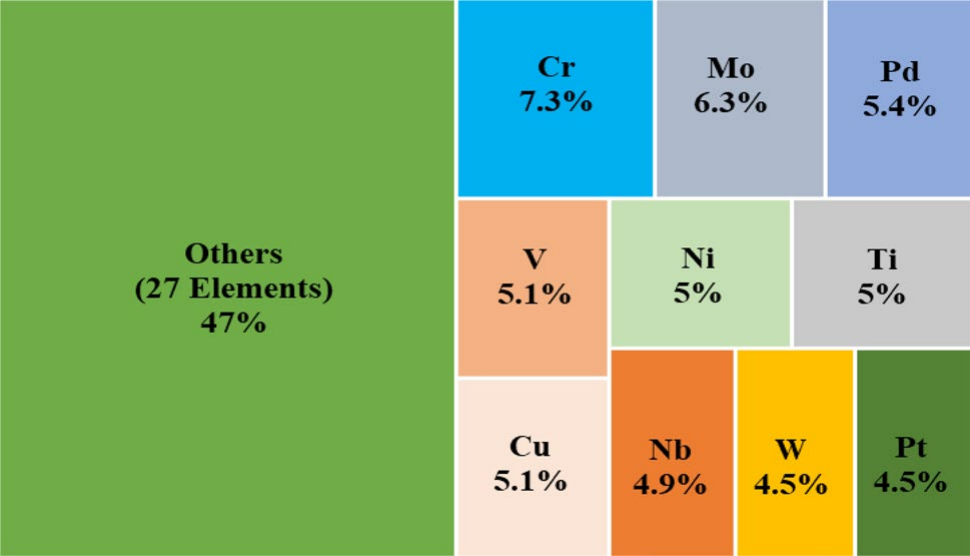
Recurrence weightage of respective elements in HEA classification dataset (total 37 elements).

An exploratory analysis tree map of the HEA classification dataset, Fig. 1, representing the recurrence of elements in the dataset reveals that more than 50% of the weightage is contributed by only 10 elements.

### Algorithm

**Figure 2:**
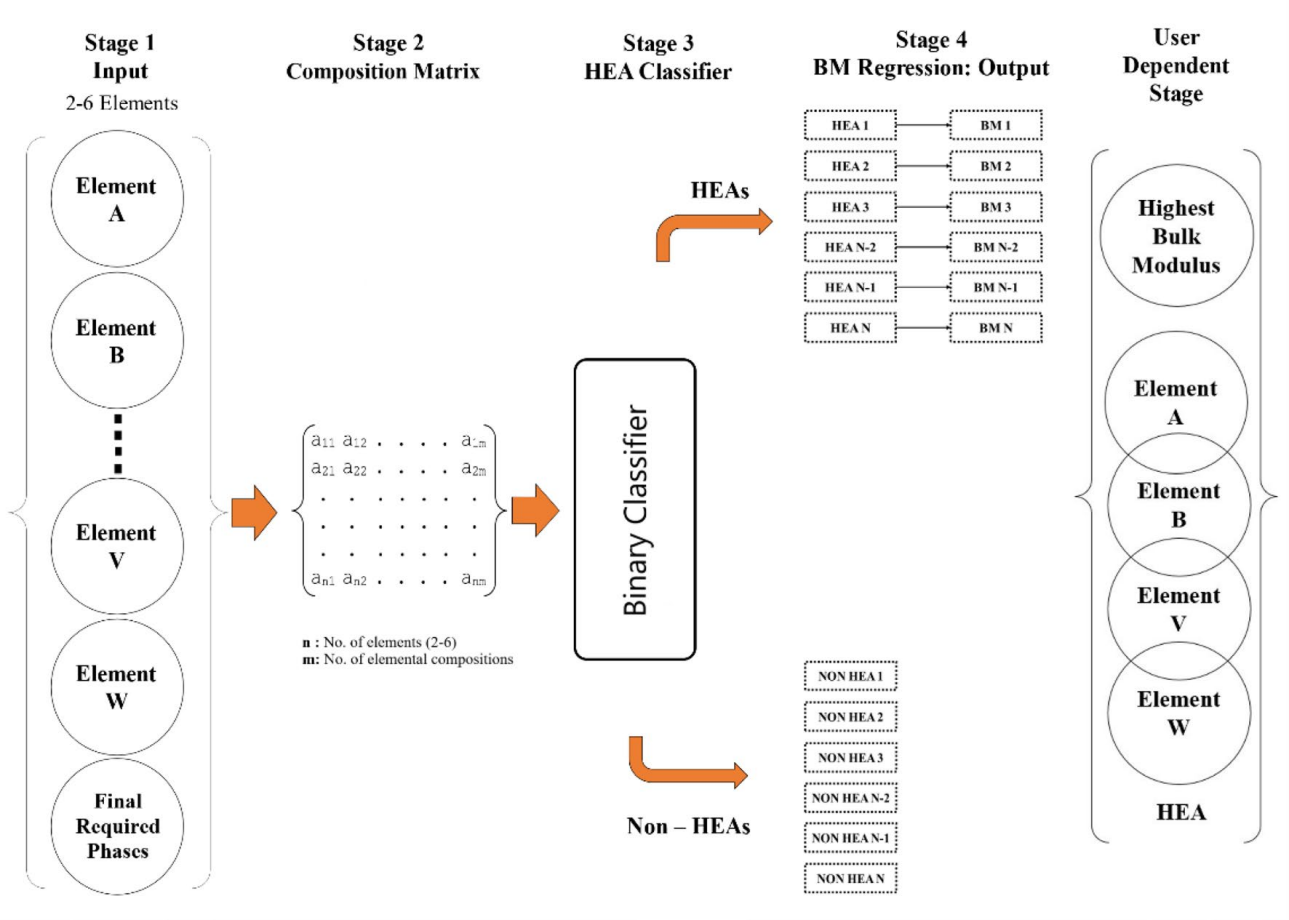
Model architecture for designing virtual HEAs.

The algorithm comprises five distinct stages, Fig. 2. In the initial stage, elements are selected from a given set along with the phase expected to be formed by the user. Calculation of all possible combinations of elemental composition for the selected elements and conversion of input phase into one-hot encoded vector, to be performed in the next stage. Followed by the classification of each elemental composition into HEA or non-HEA. For those compositions identified as HEA, predictive bulk modulus values are extrapolated. The ultimate output encompasses the elemental composition yielding the highest bulk modulus, along with the subsequent identified HEAs.

In the first stage, which is the user interface, the model takes the list of elements (2–6) from a given set along with the final required phases in the form of a binary vector as input. Five types of phases were considered in this work, 'AM', 'IM', 'SS', 'SS + IM', and 'AM + IM', of the alloy, as input parameters, where AM stands for Amorphous, IM stands for Intermetallics and SS is for Solid Solutions. For example, in the case of an alloy characterized by the 'SS + IM' phase, its binary vector will feature a "1" at the SS and IM positions, while a "0" will be assigned to the AM position (Table S18).

In the second stage, a composition matrix, which comprises all the possible combinations of elemental compositions is prepared and this in turn segregates the composition of alloys into HEA or Non-HEA clusters later. The term “combination matrix” refers to the matrix which has all possible combinations of the HEAs with varying constituent elements (2–6) ranging from 1 to 99% composition of every element. Each row of the matrix shown in Stage 2, represents a possible alloy with the selected elements.

**Figure 3:**
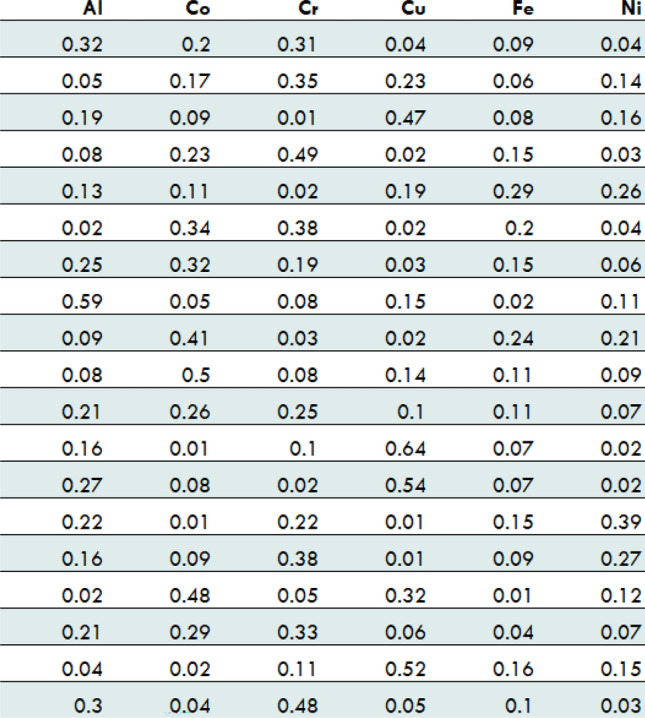
A demonstration of the composition matrix for six element alloy.

The corresponding combination matrix is selected based on the number of selected elements of the HEA. The column headings of this composition matrix are to be replaced with the names of the selected elements. This dataset is then sent as an input to the classifier model to segregate HEAs and non-HEAs. For example, a composition matrix for a HEA containing 6 elements (Fe, Ni, Co, Cr, Cu, Al) will contain 70,560,204 different compositions, only a few are shown in Fig. 3.

After the composition matrix has been built in the second stage, these values are used as inputs for the 3rd stage of the model i.e., the HEA classifier, which classifies whether the combination of selected elements and their composition are forming a HEA or not.

For demonstration and validation of HEA classifier, 32 experimental alloys (Table S16) formed by Fe, Ni, Co, Cr, Cu, and Al, were chosen from the existing dataset. The model gives ‘1’ as the output for all the 32 alloys i.e., the model classifies them as HEAs. The results were validated for these alloys from the literature and it was found that the 32 alloy compositions were in fact HEAs^31^.

If the combination does not form a HEA, then the output is shown as a non-HEA system. If the combination forms HEA, then the model moves on to the 4th stage i.e., the BM Regressor which takes the same input vector as the classifier, which results in a list of possible HEA compositions with their corresponding bulk moduli, for a given set of elements as input. The 32 HEAs were given as input to the BM regressor and the bulk modulus values were predicted.

**Figure 4:**
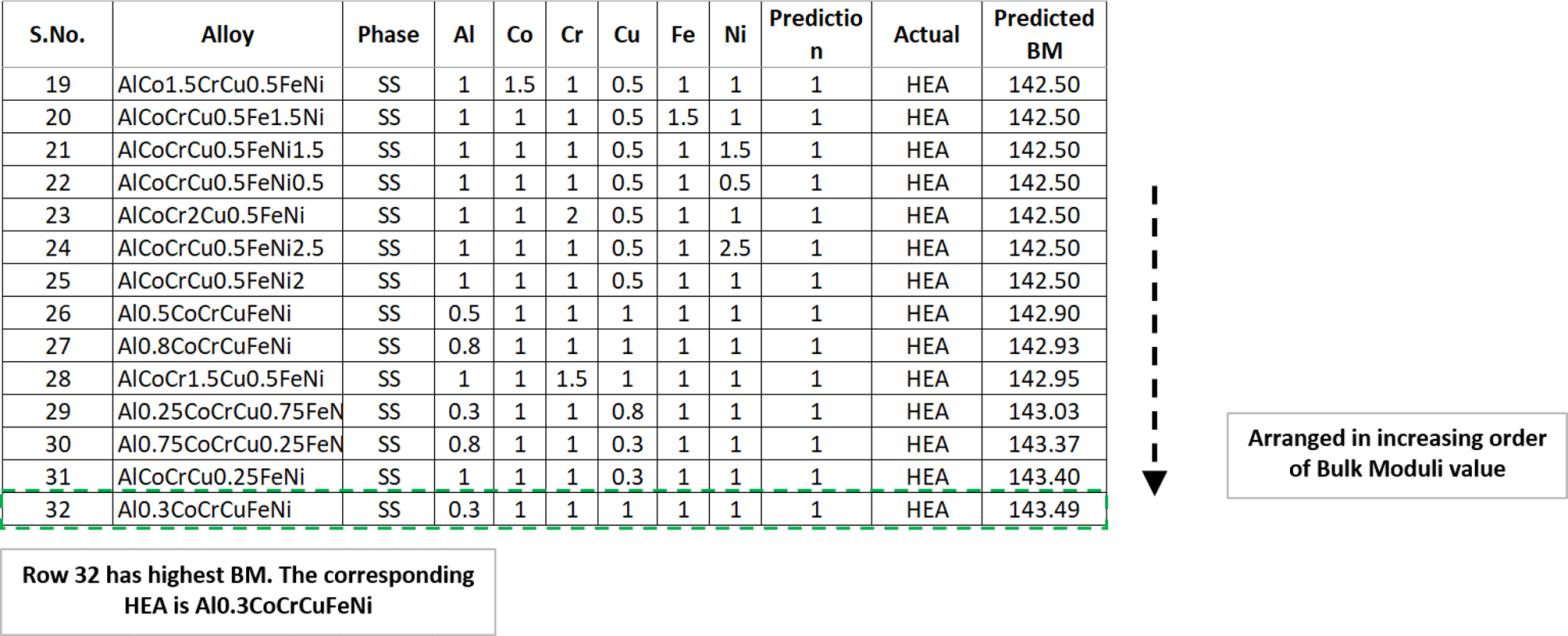
HEA configuration corresponding to their bulk modulus values.

Stage 5 is a user-dependent stage, wherein the user takes the output obtained in Stage 4 (The list of bulk modulus values), to find out the HEA composition which has the highest bulk modulus value, as shown in Fig. 4 (which is a screenshot of Table S16). To validate the BM regressor, the authors compared the experimental value and predicted value with the values available in the literature^31,32^ and the Mean Absolute Error (MAPE) obtained was 1.25%. (Table S16).

### HEA classifier

To determine the most efficient ML algorithm for HEA classification, 12 different models: Support Vector Classifier (SVC), Nu-Support Vector Classifier (Nu-SVC), XG Boost Classifier (XGB), K-Nearest Classifier (KNC), Random Forest Classifier (RFC), Extra Trees Classifier (ETC), Gradient Boosting Classifier (GBC), Adaboost Classifier (AC), Decision Tree Classifier (DTC), Logistic Regression (LR), Stochastic Gradient Descent (SGD) Classifier and Gaussian Naive Bayes Classifiers (GNB); were tested.

The performance of the models was verified using the hold-out cross-validation method in which the dataset was split into testing and training datasets for three different seeds. The dataset has been split into a 70:30 ratio, resulting in 1320 training data points and 567 testing data points (Tables S1–S6). The results of seed ‘zero’ has been presented in the work and seed ‘one’ and ‘two’ have been given in (Table S7). Apart from using the hold-out cross-validation method, to avoid overfitting over the training data, the authors have substantiated the accuracy of the model further using an internal five-fold cross-validation over the training set.

A comparative study has been conducted based on the performance of these 12 ML algorithms to determine which gives the best results. The performance of each classification model was assessed using various statistical metrics. These measures included Accuracy, which quantifies the overall correctness of predictions; Precision, indicating the proportion of true positive predictions among all positive predictions; Recall, representing the proportion of true positive predictions among all actual positive values; F1 Score, the harmonic mean of Precision and Recall to evaluate model accuracy across both classes; and Receiver Operating Characteristic-Area Under Curve (ROC-AUC) score, measuring the model's ability to distinguish between classes. These comprehensive performance metrics allowed for a robust evaluation of the classification models, which have been mentioned in Table 1 HEA Classifier.

**Table 1:** Performance results of HEA classifier based on 12 distinct ML models.

Model	Accuracy		Precision		Recall		F1_score		AUC_ROC	
	Train	Test	Train	Test	Train	Test	Train	Test	Train	Test
LR	0.71	0.69	0.73	0.66	0.56	0.54	0.63	0.59	0.70	0.67
SVC	0.87	0.78	0.87	0.76	0.83	0.70	0.85	0.73	0.87	0.77
NuSVC	0.86	0.78	0.87	0.76	0.82	0.69	0.84	0.72	0.86	0.76
SGD	0.70	0.66	0.66	0.58	0.69	0.65	0.67	0.61	0.70	0.66
KNC	0.80	0.76	0.81	0.74	0.73	0.67	0.77	0.70	0.79	0.75
GBC	0.99	0.77	0.99	0.73	0.98	0.71	0.99	0.72	0.99	0.76
AC	0.76	0.72	0.75	0.67	0.68	0.65	0.71	0.66	0.75	0.71
RFC	0.93	0.76	0.96	0.75	0.89	0.65	0.92	0.70	0.93	0.75
XGB	0.99	0.77	0.99	0.75	0.99	0.70	0.99	0.72	0.99	0.76
DTC	0.69	0.65	0.62	0.56	0.80	0.78	0.70	0.65	0.70	0.67
ETC	0.87	0.74	0.96	0.74	0.74	0.57	0.84	0.65	0.86	0.72
GNB	0.48	0.46	0.46	0.43	1.00	0.99	0.63	0.60	0.53	0.53

**Figure 5:**
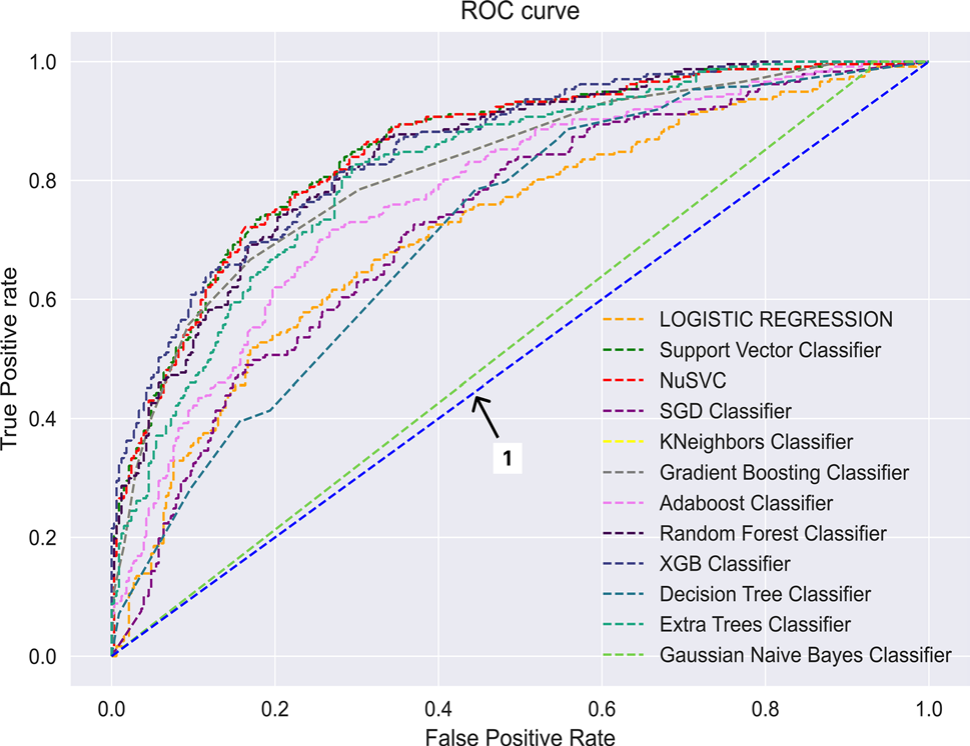
ROC Curves of Twelve HEA classifiers. Curve-1 represents a hypothetical least accurate model.

The ROC curve of all 12 classifiers for HEA classification is illustrated in Fig. 5. Generally, the area under a ROC curve determines the accuracy of the model. The larger the area, the better the performance of the model. A curve that has an area of 0.5 units i.e. a straight line is the least accurate model. Line 1 marked in the figure shows this hypothetical curve.

Greater emphasis has been placed on recall as a prime metric for inter-model performance comparison. A higher recall value guarantees the accurate identification of genuine HEAs. The Gaussian Naive Bayes (GNB) classifier attains the pinnacle in the recall, as depicted in Table 1. However, its performance significantly lags in terms of other critical metrics because of the false assumption made GNB regarding the distribution of the dataset features, as it assumes the data to be normally distributed. GBC has the second-highest recall value of 0.71. The figure illustrates that several classifiers exhibit closely aligned ROC curves, making it challenging to distinguish between them. The GBC performs on par with the other classifiers in this context. And from Table 1, it can be seen that the numerical value of this area is 0.76 units. The cross-validation scores for the HEA classifier models were also calculated and have been shared as supplementary files.

In the case of the GBC HEA classifier, the mean fivefold cross-validation score (cross_val_score) obtained for the training dataset was 0.765 with a standard deviation of 0.01 (Table S13).

To ensure consistency in classification models, two trials were done further, by splitting the train and test data into 70:30 split for random seeds 1 and 2. The accuracy from these trials as shown in Table S15, validates the consistency of GBC with test accuracies of 77.6% and 79.5% for random seeds 1 and 2 respectively. Thereby, GBC exhibits an average accuracy of 78%.

With a recall of 0.73 on the test dataset, the Gradient Boosting Classifier (GBC) model is observed to misclassify approximately 27% of HEAs as non-HEAs. This misclassification has significant implications, potentially resulting in the exclusion of HEAs with desirable Bulk modulus values from further prediction processes. Our efforts to address this issue have involved rigorous model optimization and testing across various sets of datasets. However, it's essential to note that due to the limited size and reduced generality of the dataset, further optimization attempts may risk overfitting the model to this specific dataset, and might results in in-accurate real-world testing.

### GBC model optimization

Gradient Boosting Classification (GBC) is a popular ensemble algorithm for classification in ML that aims at building a strong model by using a collection of weaker models. This idea stems from the fact that weaker models can be boosted to generate optimum results. Based on an iterative methodology, it tries to minimize the loss function by moving closer to the actual value in each iteration. GBC proved to be the most robust supervised ML algorithm out of the 12 algorithms which have been used for HEA classification, in this work. The optimization of classification metrics described above in Table 1 such as accuracy, precision, recall, F1 score, and ROC-AUC score is performed by hyper-parameter tuning using an open-source Python library Hypopt^33^. Hypopt uses a Grid Search algorithm to choose the best combination of hyperparameters after exploring various combinations of hyperparameters. The optimal configuration is shown in Table 2.

**Table 2:** Optimal configuration of GBC classifier.

Parameters	Value
Loss	Deviance
n_estimators	100
max_depth	8
random_state	0

**Figure 6:**
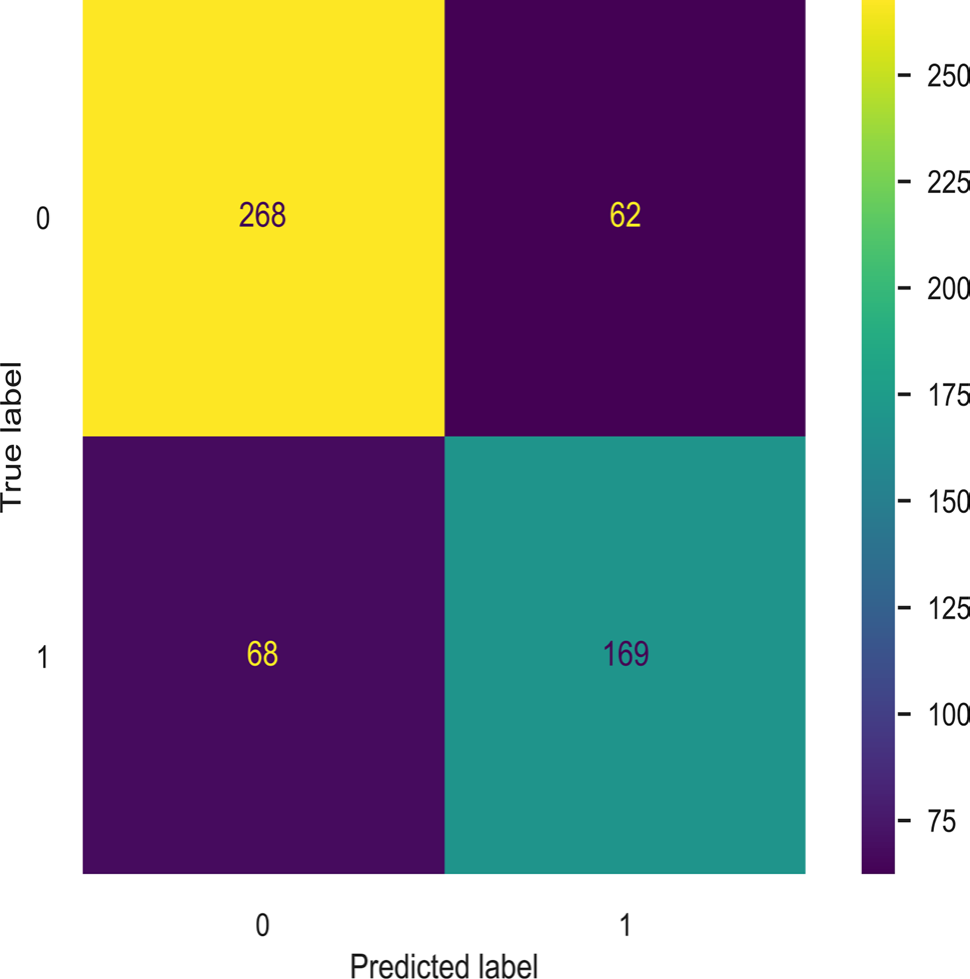
Confusion matrix for HEA classifier.

The optimised GBC model has an accuracy of 0.98 over the training set, 0.77 over the testing set, and Precision, Recall, F1 score, and ROC-AUC Score of 0.73, 0.71, 0.72, and 0.76 respectively on the test set. The GBC model gave better results than the other classical ML models comparatively to classify whether a given entry is HEA or not. To test out the occurrence of false positives and true negatives, the authors have created a confusion matrix for the 567 HEAs from test data and as shown below in Fig. 6, the classification accuracy is 77%.

### BM regressor

Regression is performed to predict the bulk modulus value of the HEA. A comparison of 7 regression models was done namely, XG Boost Regressor (XGB), Random Forest Regressor (RF), Linear Regressor, Ridge Regressor, K-Nearest Neighbour (KNN) Regressor, Elastic Net Regressor and LASSO Regressor to determine the best-performing model. A validation procedure analogous to that employed for the HEA classifier was conducted to assess the performance of the models.

The efficiency of the regression models was assessed through several statistical metrics, providing a comprehensive evaluation of the model's performance. These metrics encompass R-Square, which quantifies the proportion of variance explained by the model; Adjusted R-Square, a refined measure that considers the number of predictors; Mean Squared Error (MSE), gauging the average squared difference between predicted and actual values; Mean Absolute Error (MAE), measuring the average absolute difference between predictions and observations; and Mean Absolute Percentage Error (MAPE), offering insights into the relative accuracy of predictions. This rigorous assessment ensures a holistic understanding of the model's capabilities. Table 3 shows the values obtained for each of the regression models. As can be seen from the Table 3 the values obtained from Liner regression is negative, due to the high sparsity of the dataset and absence of any regularization method.

**Table 3:** Comparison between different BM regression model.

Model	R-square		MSE		MAE		MAPE		Adj. R-square	
	Train	Test	Train	Test	Train	Test	Train	Test	Train	Test
RF	0.99	0.86	29.71	180.41	2.43	6.55	2.36	6.95	0.97	0.83
XGB	0.99	0.90	0.07	127.42	0.05	5.46	0.03	5.88	0.99	0.88
Linear	0.99	− 4e10	1.54	6e13	0.29	4e5	0.24	5e5	0.99	− 5e10
Lasso	0.99	0.98	1.68	26.98	0.37	0.84	0.36	1.26	0.99	0.97
Ridge	0.99	0.97	1.56	30.14	0.29	0.79	0.25	0.97	0.99	0.97
ElasticNet	0.99	0.96	7.58	49.63	1.12	1.93	1.19	3.22	0.99	0.95
KNN	0.96	0.86	49.11	191.70	3.60	7.65	3.37	8.31	0.96	0.82

It is evident that Lasso Regression exhibits the highest level of accuracy in predicting bulk modulus values Table 3, with Ridge and Elastic Net Regression closely following. Least Absolute Shrinkage and Selection Operator (LASSO) Regression extends traditional linear regression by incorporating L1 regularization. This regularization technique effectively addresses multicollinearity among features and enhances feature selection. It achieves this by augmenting the ordinary least squares (OLS) cost function with a penalty term, which involves the L1 norm of regression coefficients, scaled by the regularization hyperparameter lambda (λ).

Given the high sparsity within our dataset, the feature selection capabilities of the LASSO Regressor prove to be especially advantageous in predicting the bulk moduli values of HEAs. We employed Hypopt for the fine-tuning of the lambda hyperparameter, and the optimal value yielding the most accurate results was determined to be "0.001".

**Figure 7:**
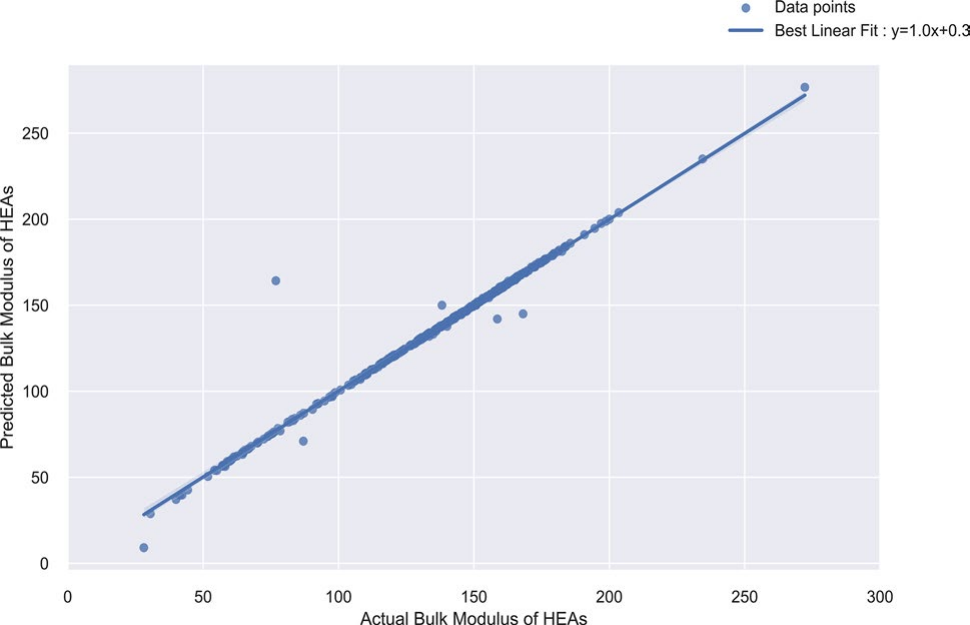
Predicted vs. actual bulk moduli for HEAs.

Predicted values of the LASSO Regressor are plotted against actual bulk modulus values, Fig. 7. Prediction values are in correlation with the actual values (experimental values reported in the literature) as shown and this is further supported by the fact that the Mean Absolute Percentage Error (MAPE) for the testing sets of HEAs is 1.26%. The proximity between R-square scores and adjusted R-square scores, i.e. 0.98 and 0.97 respectively for the LASSO regressor indicates that the model is reliable and robust. The mean five-fold cross-validation score (cross_val_score) obtained for the training dataset in the case of LASSO regression was 0.96 and a standard deviation of 0.06 (Table S14). To ensure consistency in regression models, two trials were done further, by splitting the train and test data into 70:30 split for random seeds 1 and 2. The results from these trials as shown in Supplementary File 17, validates the consistency of the LASSO Regressor, with an average test R^2^ value of 0.991.

## Conclusion

As demonstrated in this work Machine Learning models such as the GBC classifier and LASSO Regressor have a huge potential in determining the set of HEAs with optimum bulk modulus values. These predicted bulk moduli assist the user in selecting a HEA corresponding to the highest bulk modulus for high-strength applications. This reduces the search space of experimentalists in successfully fabricating a high bulk moduli HEAs. Further, this study can be extended to determine the correlation between other properties of HEAs based on their elemental composition. Designing virtual HEAs using ML models reduces the reliance on conventional experimental methods, and hence acts as an efficient alternative to them. As there’s an increase in the number of datasets, the accuracy of these models can be increased multi-folds.

### Supplementary Information

Supplementary Table S1.

Supplementary Table S2.

Supplementary Table S3.

Supplementary Table S4.

Supplementary Table S5.

Supplementary Table S6.

Supplementary Table S7.

Supplementary Table S8.

Supplementary Table S9.

Supplementary Table S10.

Supplementary Table S11.

Supplementary Table S12.

Supplementary Table S13.

Supplementary Table S14.

Supplementary Table S15.

Supplementary Table S16.

Supplementary Figures.

Supplementary Table S18.

## Data Availability

The processed data and supplementary required to reproduce these findings are available to download from https://github.com/anshpoonia/Design-of-High-Bulk-Moduli-High-Entropy-Alloys-using-ML.git.
