# Supplementary material for: Design of high bulk moduli high entropy alloys using machine learning
Source: Sci Rep. 2023 Nov 22;13:20504. doi: 10.1038/s41598-023-47181-x (PMC10665368; doi:10.1038/s41598-023-47181-x)
Supplement: Supplementary file 17 — Supplementary Figures. [file 41598_2023_47181_MOESM17_ESM.docx]

**Supplementary File 16. Predicted vs. Actual Bulk Modulus Values for Random Seeds**


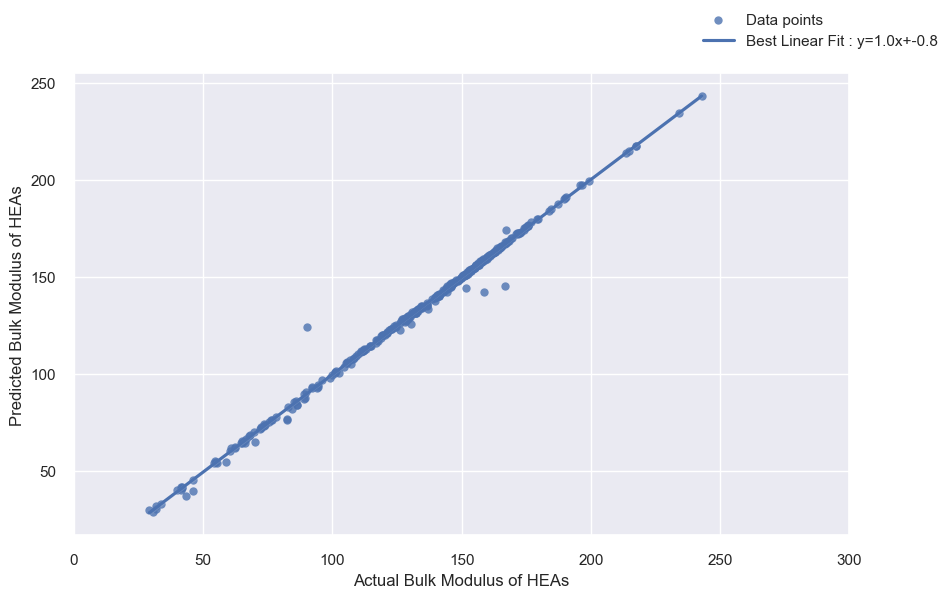


**Figure S1.** Predicted vs. Actual Bulk Modulus Values for Random Seed – 1


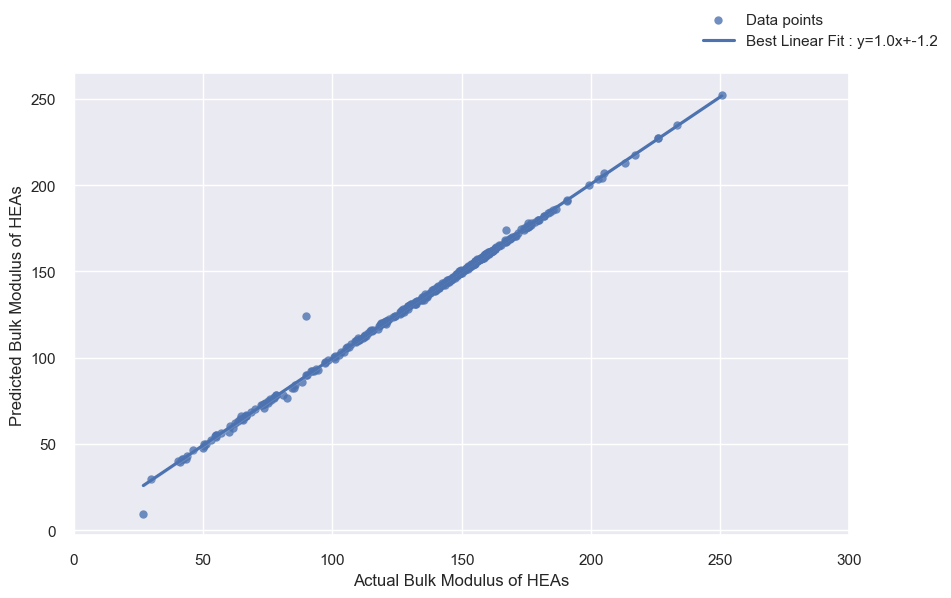


**Figure S2.** Predicted vs. Actual Bulk Modulus Values for Random Seed – 2
